# Dual-Edged Character of Quorum Sensing Signaling Molecules in Microbial Extracellular Electron Transfer

**DOI:** 10.3389/fmicb.2018.01924

**Published:** 2018-08-20

**Authors:** Yang Yang, Huihui Zhou, Xiaoxue Mei, Bingfeng Liu, Defeng Xing

**Affiliations:** State Key Laboratory of Urban Water Resources and Environment, School of Environment, Harbin Institute of Technology, Harbin, China

**Keywords:** quorum sensing, autoinducers, extracellular electron transfer, microbial fuel cell, biofilm

## Abstract

Quorum sensing (QS) is a central mechanism for regulating bacterial social networks in biofilm via the production of diffusible signal molecules (autoinducers). In this work, we assess the contribution of QS autoinducers to microbial extracellular electron transfer (EET) by *Pseudomonas aeruginosa* strain PAO1 and three mutants pure culture-inoculated in microbial electrolysis cells (MECs) and microbial fuel cells (MFCs). MECs inoculated with different *P. aeruginosa* strains showed a difference in current generation. All MFCs reached a reproducible cycle of current generation, and PQS-deficient *pqsA* mutant inoculated-MFCs obtained a much higher current generation than *pqsL* mutant inoculated-MFCs which overproduced PQS. *lasIrhlI*-inoculated MFCs produced a lower power output than others, as the strain was deficient in *rhl* and *las*. Exogenous N-butanoyl-l-homoserine lactone could remedy the electricity production by *lasIrhlI* mutants to a level similar to wild-type strains while signaling molecules had little effect on wild-type bacteria in MFCs. Meanwhile, experiments with the wild-type and *pqsA*, *pqsL* mutants indicated that the overexpression of PQS signaling molecules made no significant contribution to EET. QS signaling molecules therefore have dual-edged effects on microbial EET. These findings will provide favorable suggestions on the regulation of EET, but detailed QS regulatory mechanisms for extracellular electron transfer in pure- and mixed-cultures are yet to be elucidated.

## Introduction

Microbial electrochemical systems (MESs) are a versatile group of technologies with the potential to achieve sustainable bioenergy generation, biosensing and bioelectrosynthesis using organic or inorganic carbon sources (Liu et al., 2016). Electroactive bacteria (EAB) function as biocatalysts and are able to exchange electrons between cells and electrodes via multiple processes of extracellular electron transfer (Bond and Lovley, 2003; Rabaey et al., 2004; Reguera et al., 2005). The optimal reactor configurations, operating conditions and electrode materials for increased electron transfer in MES have been described previously (Rinaldi et al., 2008; Karthikeyan et al., 2015; Logan et al., 2015; Mei et al., 2015; Xing et al., 2015; Zou et al., 2016; Bi et al., 2018). However, further improvement of electron transfer in MES is difficult due to inadequate understanding of electrode-biofilm formation in EAB. Low efficiency electron transfer at the anodic biofilm-electrode interface remains one of the major limitations for full-scale implementation of microbial fuel cells (MFCs) (Logan, 2009). Manipulating exoelectrogenic biofilms to improve the efficiency of the electron transfer pathway is therefore a feasible strategy to improve MES performance.

Quorum sensing (QS) is a cell-cell communication mechanism in which extracellular signal molecules called autoinducers are released by bacteria. The autoinducers pass on information about population density, and the bacteria population then collectively regulates the expression of related genes as a response (Swift et al., 1996). QS has been found to regulate many bacterial physiological activities, including biofilm formation, in several bacterial species (Davies et al., 1998; Zhu and Mekalanos, 2003; Boles and Horswill, 2008). In particular, relatively complex QS systems are found in *Pseudomonas aeruginosa* (Juhas et al., 2005), a species commonly found in the biofilms of MESs. The *las* system of QS in *P. aeruginosa* is comprised of the transcriptional activator LasR and the N-(3-oxododecanoyl)-L-homoserine lactone (3-oxo-C12-HSL) signal molecule synthase LasI (Gambello and Iglewski, 1991; Passador and Iglewski, 1993). Similarly, in the *rhl* system, the enzyme RhlI catalyzes the synthesis of the N-butyryl-L-homoserine lactone (C4-HSL) signal molecule, which is detected by the transcriptional activator protein RhlR (Ochsner et al., 1994; Parsek et al., 1997). In addition to the two N-Acyl homoserine lactone (AHL) type signal molecules, a third autoinducer, 2-heptyl-3-hydroxy-4-quinolone (designated as *Pseudomonas* quinolone signal, PQS), provides a link between the *las* and *rhl* quorum-sensing systems (Pesci et al., 1999; Mcknight et al., 2000). These QS systems constitute a hierarchical regulation network in *P. aeruginosa* (Williams and Camara, 2009; Yong et al., 2015).

Recent studies have shown that QS signaling molecules play crucial roles in electricity generation by MFCs (Yong et al., 2011, 2015; Chen et al., 2017). For example, genetic enhancement of the QS circuit was applied in MFCs to enhance electricity production (Yong et al., 2011). The overexpression of *rhlI* and *rhlR* genes in wild-type strain lead to a significant increase in phenazine production, which directly resulted in an increase of current output in the *rhlI* overexpressed strain inoculated-MFCs. A PQS defective strain also produced higher concentrations of phenazines and exhibited increased current production when used in MFCs compared to the parent strain (Wang et al., 2013). Electrochemical activity of the bio-anode was promoted by the addition of 3-oxo-hexanoyl-homoserine lactone and 3-oxo-dodecanoyl-homoserine lactone in microbial electrolysis cells (MECs), and a higher current was produced with the addition of short chain acyl-homoserine lactone (Liu et al., 2015). In particular, these studies substantiated the observation that QS regulatory networks are involved in microbial extracellular electron transfer, and most recent studies focused on genetically engineering QS to improve the electricity output in MFCs. However, it is still unclear which signal molecules could be applied to improve MFC performances, and whether or not they could remedy the electricity production of mutants. In this study, *P. aeruginosa* strain PAO1 and its three mutants were pure culture-inoculated in MFCs and MECs to investigate the effect of QS on extracellular electron transfer. We subsequently assess the ability of QS to enhance attachment of anodic bacteria and current generation of MFCs by the addition of exogenous QS signals such as PQS and AHLs.

## Materials and Methods

### Bacteria and Culture Conditions

*Pseudomonas aeruginosa* PAO1 (wild-type) and three mutants (*pqsA*, *pqsL*, and *lasIrhlI*) were kindly provided by Tim Tolker-Nielsen (University of Copenhagen). The *pqsA* mutant was deficient in the production of PQS signal molecule while *pqsL* mutant overproduced PQS. The *lasIrhlI* mutant was deficient in *rhlI* and *lasI*, and so produced fewer AHL autoinducers (Allesen-Holm et al., 2006). All strains were routinely cultivated in Luria–Bertani (LB) medium with shaking at 37°C. 0.5 mL of overnight cultures were inoculated in 50 mL fresh LB medium for subsequent inoculation in single-chamber MFCs and MECs. The optical density at 600 nm (OD_600_) of incubated cultures was measured and all cultures were adjusted to the same level before inoculation.

### MFC and MEC Configuration and Operation

**FIGURE 1:**
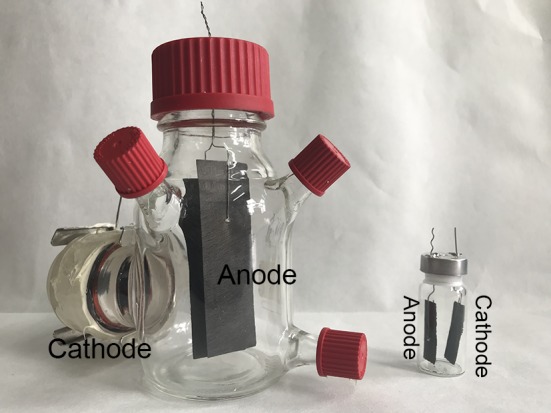
Photographs of air-cathode microbial fuel cell (left) and microbial electrolysis cell (right) containing carbon paper electrodes.

Eight single-chamber air-cathode MFCs (**Figure 1**) were constructed using glass bottles as described in previous studies (Logan et al., 2007). Two pieces of carbon paper anode (2.5 cm by 8 cm; TGP-H090, Toray, Japan) were connected to titanium wires and then tied together as a whole. The anode was fixed through a hole in the bottle lid, sealed with silicone (GE83, GE Toshiba Silicones, Japan) and suspended completely in the medium. The reactors were covered by aluminum foil prior to autoclave. Air-cathodes (7 cm^2^) were fabricated by rolling activated carbon with polytetrafluoroethylene (PTFE) to form a catalyst layer, according to a previous study (Dong et al., 2012). After assembly, the whole reactors were packed in sterilizing bags and autoclaved at 0.1 MPa, 121°C for 15 min and an air filter membrane (0.22 μm; FGLP04700, Merck Millipore, Germany) was added outside of the air cathodes. MFCs were fed with 300 mL of medium (after sterilization) containing (per liter) 1 g sodium acetate in 100 mM phosphate buffer solution (PBS), metal salt (12.5 mL) and vitamin solutions (5 mL). 5 mL of bacterial culture solution (OD_600_ = 0.5) was added into the reactors as inoculum. All reactors operated in fed-batch mode in a temperature-controlled room at 35°C.

Eight single-chamber MECs (**Figure 1**) were constructed using 5 mL glass bottles as reported previously (Call and Logan, 2011). Carbon paper (2.5 cm by 1 cm) and rolling activated carbon (2.5 cm by 1 cm) were adopted as anode and cathode, respectively. After assembly, the whole reactors were autoclaved and fed with 5 mL of medium as described above. 0.5 mL of bacterial culture solution (OD_600_ = 0.5) was added into the reactors as inoculum. The fixed voltage of 0.8 V was applied to the whole cells by connecting the positive pole of a programmable power source (3645A, Array, Inc.) to the anodes, and the negative to the cathodes. All reactors were operated in a temperature-controlled room at 35°C.

The voltage across an external resistor of 1 kΩ (MFC) and 10 Ω (MEC) was automatically monitored through a data acquisition system (2700, Keithley Instrument, United States) connected to a personal computer. The current density was calculated as previously described (Logan et al., 2006).

Two AHLs type signal molecules of 3-oxo-C12-HSL, C4-HSL and PQS were purchased from Sigma-Aldrich (United States). Signal molecules were dissolved in dimethyl sulfoxide (DMSO) and sterilized with filter membrane (Millex-GP, 0.22 μm, Merck Millipore, Germany). These autoinducers were added with final concentration levels of 10 μM as previously described (Valle et al., 2004; Monzon et al., 2016).

### Biomass Measurements

Anodic biofilms and cell suspension were collected from MFCs for protein extraction by ultrasound-assisted alkali pretreatment (Tian et al., 2015). Protein concentration was measured using a modified BCA Protein Assay Kit (Sangon Biotech, China). The effect of signal molecules on bacterial growth was assessed by pure culturing bacteria in 96-well plates (triplicates). 50 μL (OD_600_ = 0.5) bacterial culture and 350 μL sodium acetate (1 g/L) were added into the 96-well plates. Signal molecules were added separately to make a final concentration of 10 μM. An equivalent amount of DMSO was added to control cultures. The bacterial growth curve was acquired by using Bioscreen C automatic growth analyzer (Lab systems, Finland).

## Results and Discussion

### Electrochemical Performance of Pure-Culture MECs

**FIGURE 2:**
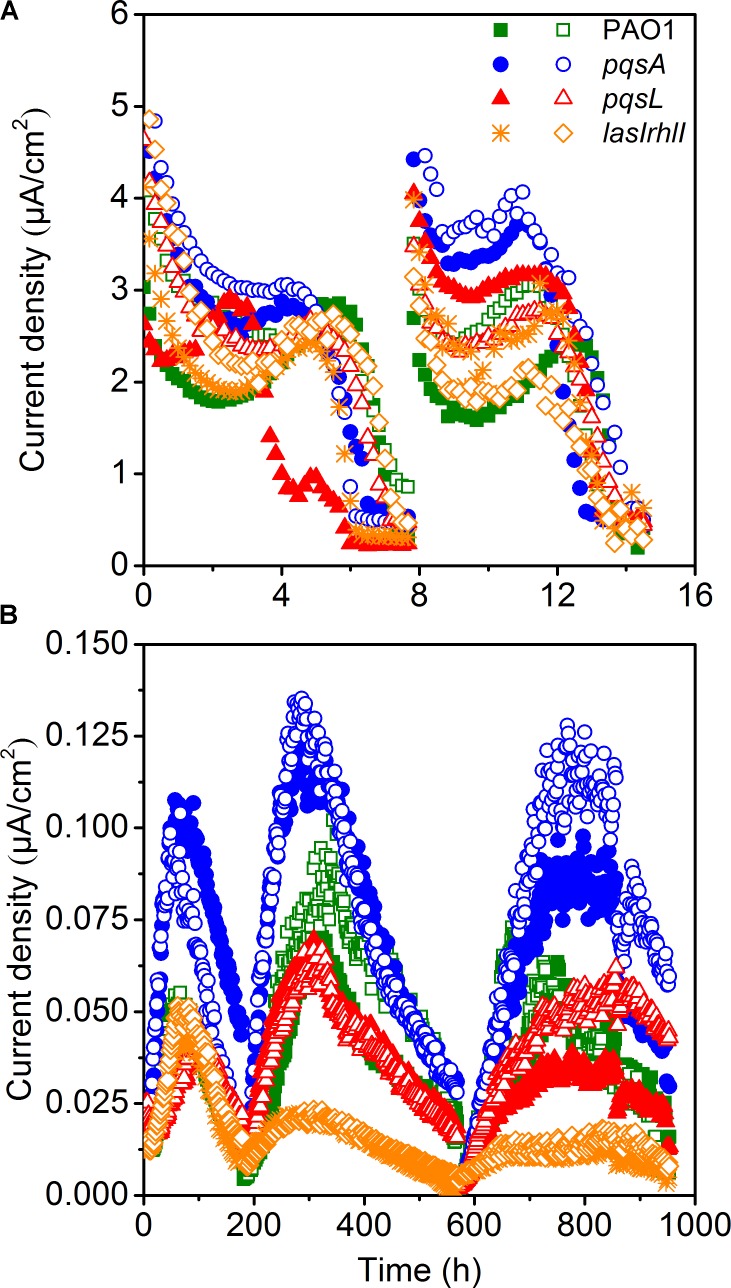
Current generation of MECs **(A)** and MFCs **(B)** with the strains of PAO1 and three mutants.

The small-scale MECs could provide high throughput operation to maintain pure culture conditions. MECs inoculated with different *P. aeruginosa* strains showed a difference in current generation (**Figure 2A**). Two cycles of current generation indicated that the current output of the *pqsA* strain with deficient production of PQS was higher than that of *pqsL* with overproduced PQS. The overexpression of PQS signaling molecules unexpectedly limited extracellular electron transfer. In comparison to the wild-type, the AHL-deficient *lasIrhlI* mutant showed lower EET (extracellular electron transfer) rate based on the average of duplicate reactors.

### Electrochemical Performance of Pure-Culture MFCs

Microbial fuel cells inoculated with four different strains of *P. aeruginosa* were operated simultaneously to further investigate the difference in electricity generation. MFC tests showed a similar and more obvious result compared to MECs. All MFCs attained reproducible cycles of current generation (**Figure 2B**). The maximum current density generated by wild-type *P. aeruginosa* PAO1 in MFC was 0.105 μA/cm^2^. It is at a similar level as a previous study with the same PAO1 strain, which generated approximately 0.150 μA/cm^2^ in a MFC (Wang et al., 2013). The coulombic efficiency was relative low, as it was not higher than 5%. Although the current generation and electron recovery from substrate was low, we could still analyze the effect of QS on the EET clearly. MFCs inoculated with *pqsA* mutants obtained the highest current density. The *lasIrhlI*-inoculated MFC produced lower current density than the other strains, due to its deficiency in *rhlI* and *lasI*. The AHLs QS systems induced cell lysis and extracellular DNA release which were responsible for biofilm structure formation in *P. aeruginosa* strains (Allesen-Holm et al., 2006). Additionally, the production of pyocyanin, which functions as an electron shuttle to enhance electron transport (Rabaey et al., 2005), is highly regulated by *las* and *rhl* (Brint and Ohman, 1995; Latifi et al., 1995; Pesci et al., 1997). The *rhl* QS system directly affect the EET as the production of pyocyanin was overproduced by the overexpression of the *rhl* genes (Yong et al., 2011). While the *las* QS system seemed to make indirect effect on the synthesis of pyocyanin, as it controls the *rhl* QS in two ways (Pesci et al., 1997). Thus, the susceptible biofilm (Allesen-Holm et al., 2006; Sakuragi and Kolter, 2007) and reduced production of pyocyanin may lead to the observed decrease in current generation of *lasIrhlI*-inoculated MFCs. The MFCs inoculated with PQS-deficient *pqsA* mutants obtained a much higher level of current generation than those inoculated with *pqsL* mutant, which overproduced PQS. PQS was demonstrated to inhibit bacteria growth (Toyofuku et al., 2008, 2010). Thus, large amount of PQS overproduced by the *pqsL* mutant might repress the production of electron shuttles, which limited the EET from EAB to the anode. However, to understand the regulation mechanism needs the more evidences of biofilm formation and transcriptome in the future. Conversely, PQS-deficient *pqsA* mutants could produce normal concentration level of electron shuttles under the directly regulation of *rhl* QS system and avoid negative effect of PQS, which promoted the EET with anode. Therefore, comparable experiments with the wild-type and mutant strains indicated that the overexpression of signaling molecules may limit the current generation in MFCs.

The difference in performance between the MEC and MFC was mainly due to differences in operating conditions. The configuration is an important factor that could affect the performance of MFCs (Logan et al., 2015) and MECs (Kadier et al., 2016). Previous studies suggest that MFCs fed with acetate as substrate produce higher power densities in small scale reactors in comparison to large-scale (Logan et al., 2015). In this study, the MFC volume (300 mL) was 60 folds larger than the MEC volume (5 mL). The MEC had a higher specific anode surface area (100 m^2^/m^3^) than the MFC (24.2 m^2^/m^3^). In addition, a higher ratio of inoculation was used in the MEC (10%) in comparison to the MFC (1.5%). Although the MEC could have higher current density and lower reagents costs because of its small size, the limited growth of *P. aeruginosa* under anaerobic conditions (Toyofuku et al., 2008) and insufficient biofilm formation resulted in the MEC being unable to be operated as long as the MFC. The oxygen level was also an important factor for the PQS system (Schertzer et al., 2010). Since oxygen was limited, the differences between different strains was not as visible in the MEC.

### Effects of Biomass on Current Generation

**FIGURE 3:**
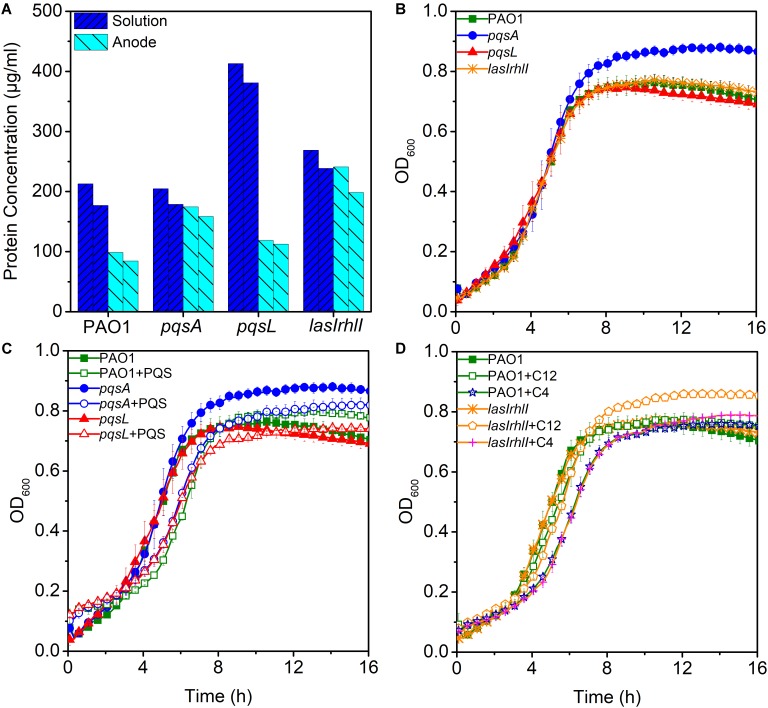
The maximum and minimum measured biomass of anode and solution in MFCs **(A)**. Bacterial growth curves without signal molecules **(B)** and with PQS **(C)** and AHLs **(D)**. Error bars represent the standard deviation of triplicate **(B**–**D)** tests.

Low production of currents in MFCs may be due to less enriched bacteria on the anode. To evaluate the hypothesis that anodic biomass may affect the current output, a measurement of protein concentration was made for biomass estimation during the third batch of MFC operation. Biomass of anode and solution in MFC were analyzed, respectively. Despite the highest anodic biomass, the *lasIrhlI*-inoculated MFC still had lower performance in electricity production (**Figure 3A**), while, the *pqsL*-inoculated MFC had a higher biomass in solution than other groups. This is largely due to the elevated release of extracellular DNA, as the QS-regulated DNA release can cause cell lysis (Webb et al., 2003). These results indicate that anodic biomass did not significantly affect the electricity production compared to QS.

### Effects of QS Signals on Bacterial Growth

To examine the effect of different signal molecules on the growth of *P. aeruginosa*, the bacteria were grown in 96-well plates and signal molecules were added separately until a final concentration of 10 μM was reached. First, we examined the growth curve of different strains. There were no significant differences between them, except for *pqsA* which had a higher stationary phase OD than other strains (**Figure 3B**). When PQS was added to the cultures of PAO1, *pqsA*, and *pqsL*, a longer lag phase was observed. In addition, the *pqsA*+PQS had a shorter stationary-phase OD than *pqsA* (**Figure 3C**). These results indicate that PQS suppressed cell density and affected bacterial growth, as has been observed previously (Häussler and Becker, 2008; Toyofuku et al., 2008). Furthermore, the exogenous addition of 3-oxo-C12-HSL and C4-HSL increased the stationary phase OD of the *lasIrhlI* mutant strain (**Figure 3D**). The accumulation of extracellular DNA in the late-log phase was restored in the *lasIrhlI* mutant culture supplemented with signal molecules in a previous study (Allesen-Holm et al., 2006), which indicated that AHL QS regulated bacterial growth. The addition of signal molecules did not significantly influence the growth of the wild type planktonic cultures.

### Effects of QS Signaling Molecules on the Performance of Pure-Culture MFCs

**FIGURE 4:**
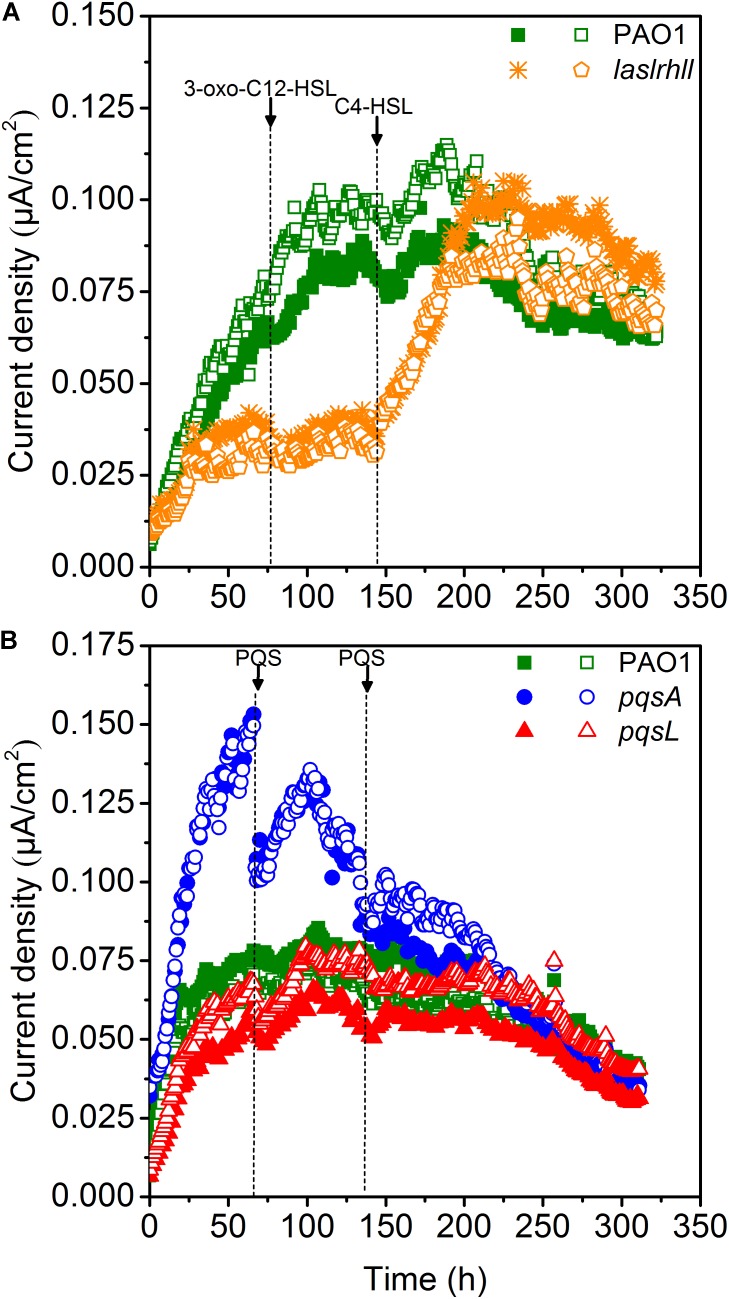
Current generation of MFCs after the addition of AHLs **(A)** and PQS **(B)**. Arrows indicate the time point for adding QS signal molecules.

Quorum sensing played important roles in mediating the bioelectrochemical characteristics in MFCs. The pyocyanin-mediated EET from EAB to the anode is crucial for electricity generation in *Pseudomonas*-based MFC and the biosynthesis is regulated by QS systems (*las*, *rhl*, and PQS) (Rabaey et al., 2005; Yong et al., 2011; Wang et al., 2013). The electron shuttle biosynthesis is directly regulated by the *rhl* QS system as the overexpression of *rhl* QS system could lead a significant improvement in electricity generation (Yong et al., 2011). While the *las* QS system seemed to make indirect effect on the synthesis of pyocyanin, as it controls the *rhl* QS system and the mutations of *las* QS system decreased the pyocyanin production (Pesci et al., 1997). Besides, the PQS signaling system also regulates electron shuttle biosynthesis but this regulatory network may be inactivated due to the oxygen level (Schertzer et al., 2010). In order to further investigate the effect of QS on extracellular electron transfer regulation, the effect of the AHLs signal molecules on EET of wild-type and *lasIrhlI* mutant strains was studied in single-chamber air-cathode MFCs. 3-oxo-C12-HSL and C4-HSL were added until final concentration levels of 10 μM were reached. Results showed that adding 3-oxo-C12-HSL signal molecule caused no significant increase in EET of either wild-type or mutant strains, indicating that the *las* QS system may not directly affect EET in MFCs (**Figure 4A**). Although it showed that the *las* QS system could make indirect effect on the synthesis of pyocyanin by controlling the *rhl* QS system in *P. aeruginosa*, the *rhl* QS system could not be activated in the *lasIrhlI* mutant due to the deficiency of *rhlI* gene. However, a very significant increase was caused in *lasIrhlI* inoculated MFCs by the addition of C4-HSL signal molecule. This could increase the electricity production of mutant strains to a level similar to wild-type strain. In a previous related study, the engineered strains could attain much higher current than the wild-type parent strains by overexpressing the *rhl* QS system. This genetic manipulation resulted in an enhanced transcription level of the *rhlI* and *rhlR* genes, which increased the amount of signal molecules and transcriptional activators simultaneously and strengthened the current production when compared with wild-type (Yong et al., 2011). Thus, separately compensating for C4-HSL signal molecule in *lasIrhlI* mutant-inoculated MFCs restored the current generation to normal levels. Meanwhile, both signal molecules had little effect on wild-type strain-inoculated MFCs.

Microbial fuel cells inoculated with PAO1, *pqsA* and *pqsL* were supplemented with 10 μM of PQS. The maximum current decreased after adding PQS to *pqsA*-inoculated MFCs (**Figure 4B**). However, there was no significant effect on wild-type and *pqsL* mutants. In *P. aeruginosa*, PQS decreased growth rates under aerobic conditions and repressed anaerobic growth (Häussler and Becker, 2008; Toyofuku et al., 2008). Previous studies showed that *P. aeruginosa* didn’t produce detectable PQS anaerobically (Toyofuku et al., 2008; Schertzer et al., 2010). However, the special configuration of the air-cathodes formed a relatively micro-aerobic environment in the MFCs. Thus, the wild-type and *pqsL* mutants could both produce a considerable amount of PQS, which had negative effects on MFC performance. The addition of exogenous PQS to higher performance MFCs (*pqsA*-inoculated) decreased the current generation. This result is similar to a previous study (Wang et al., 2013), in which a PQS defective mutant inoculated MFC attained the lowest current generation. We hypothesized that this difference was likely caused by differences in reactor configuration. A higher oxygen level was available in the air-cathode MFCs than in dual chamber U-tube MFCs, which promoted bacterial growth (Toyofuku et al., 2008) and pyocyanin biosynthesis (Schertzer et al., 2010).

## Conclusion

In summary, we investigated the effect of signal molecules on MFC performance and found that C4-HSL could increase the current generation of gene deficient variants to that of unmodified strains. PQS quorum sensing made no significant contribution to current output, and anodic biomass did not significantly alter electricity production when compared with QS. Thus, strategies to up-regulate the *rhl* QS system while limiting the PQS production are adoptable. However, the exogenous addition of signal molecules had little effect on the normal strain, which indicated that more efficient manipulation and utilization of QS needs to be further explored in mixed cultures MFCs. These findings will help provide suggestions for the enhancement of EET in MESs.

## Author Contributions

DX designed the experiments. YY performed the specific experiments. YY, HZ, XM, BL, and DX contributed to analyze the experiment data. YY and DX wrote the manuscript. All authors were involved in revision of the manuscript and approved the final manuscript.

## Conflict of Interest Statement

The authors declare that the research was conducted in the absence of any commercial or financial relationships that could be construed as a potential conflict of interest.
